# Anti-tumor effects of CIK combined with oxaliplatin in human oxaliplatin-resistant gastric cancer cells in vivo and in vitro

**DOI:** 10.1186/1756-9966-29-118

**Published:** 2010-08-30

**Authors:** Qun Zhao, Hui Zhang, Yong Li, Jun Liu, Xiaojie Hu, Liqiao Fan

**Affiliations:** 1 Department of Surgery, The Fourth Hospital of Hebei Medical University, Shijiazhuang 050011, China; 2 Department of Gynecology, The Fourth Hospital of Hebei Medical University, Shijiazhuang 050011, China

## Abstract

**Background:**

Drug resistance remains a great challenge in the treatment of gastric cancer. The goal of this study was to explore the anti-tumor effects and mechanism of cytokine-induced killer (CIK) cell combined with oxaliplatin (L-OHP) in human oxaliplatin-resistant gastric cancer cells.

**Methods:**

After producing oxaliplatin-resistant gastric cancer cells, cell morphology, growth and doubling time were observed, followed by detection of cell cycle distribution and apoptosis, drug sensitivity (e.g., L-OHP) and expression of P-gp and livin. MTT assay, in vivo pharmacodynamics and pathomorphology experiments were used to detect killing activities of CIK combined with L-OHP.

**Results:**

Compared with parental gastric cancer cells, oxaliplatin-resistant gastric cancer cells in S phase were reduced and cell apoptosis rate was increased (P < 0.05), the inhibition rate of 10 chemotherapeutics on oxaliplatin-resistant gastric cancer cells was significantly lower and the expression of P-gp was significantly higher (P < 0.05). However, there was no significant difference in livin expression between parental gastric cancer cells and oxaliplatin-resistant gastric cancer cells (P > 0.05). The in vitro killing activity of CIK combined with L-OHP on parental cells and oxaliplatin-resistant cells were significantly enhanced compared with L-OHP or CIK alone. And it showed greater synergetic effects against oxaliplatin-resistant cells compared with parental cells (P < 0.05). In addition, survival rate, abdominal circumference and pathomorphology results revealed stronger in vivo anti-tumor effects when the two therapies were combined.

**Conclusions:**

The mechanism of oxaliplatin-resistant cell secondary multidrug resistance was correlated with the variation of cell cycle distribution, extension of doubling time and upregulation of P-gp expression. The synergistic effect of CIK in combination with L-OHP on killing activity against oxaliplatin-resistant cells was shown in vivo and in vitro.

## Background

In addition to surgery, chemotherapy is the most effective adjuvant therapy for recurrent and metastasized malignant tumors. Although current chemotherapy programs continue to improve, individual differences of tumor patients in the sensitivity to chemotherapeutics have led to chemotherapeutic efficacy in most patients remaining unsatisfactory. Even within an individual, the same drug can have differing effects during different stages of cancer. Multidrug resistance (MDR) is considered as one of the main disturbances affecting chemotherapeutic effects. Drug-resistant protein that induces MDR was always over-expressed within medication, shown to render chemotherapeutics unable to enter the effector target (i.e., the nucleus), leading to the failure of chemotherapy.

Currently, platinum family is the powerful chemotherapy drug widely used in clinical. Cisplatin (CDDP) showed excellent therapeutic effects on various tumors in several organs, including lung, ovary, bladder, pate, esophagus, cervix, endometrium and testis [1]. Additionally, oxaliplatin (L-OHP) was regarded as a third generation novel type of platinum compounds following CDDP and carboplatin, replacing the amino group of cisplatin with a bulky diaminocyclohexane (DACH) ring [2] and showing specific properties of high efficiency and low toxicity [3,4]. Moreover, L-OPH was shown to be effective in primary CDDP- and carboplatin-resistant colon carcinoma and some secondary CDDP-resistant malignant tumors [5-7]. Gastric cancer is a common alimentary canal malignant tumor, which shows both primary and secondary drug resistance. Chen et al. considered that the drug-resistant mechanisms of gastric cancer to L-OHP and CDDP were correlated with augmentation of DNA repair and ATP7A overexpression [8]. MDR mechanisms of gastric cancer cells were detected to aid in choosing effective anti-cancer drugs, and individualized treatment plans were made, resulting in improved gastric therapeutic effects.

With the rapid developments in the field of tumor immunology, use of immune effector cells, including lymphokine-activated killer (LAK), tumor-infiltration lymphocyte (TIL), anti-CD3 antibody induced activated killer (CD3AK) and cytolytic T lymphocyte (CTL) cells, on certain advanced-stage tumors has shown therapeutic effects [9], and this treatment could kill remnant chemotherapy-resistant tumor cells [10]. Cytokine-induced killer (CIK) cells are a novel type of immunocompetent cells with highly efficient and broad-spectrum anti-tumor activity. These cells have been shown to proliferate among and directly kill CD3^+^CD56^+ ^tumor cells in vitro [11-13]. Furthermore, CIK cells were shown to enhance cellular immune function in hosts [14,15], and previous studies showed the killing activity of CIK cells on MDR tumor cells was similar or greater than that on parental drug-sensitive tumor cells [16,17]. This treatment is thought to be effective for patients with recurrent tumors when combined with chemotherapy [10,18-20].

In this study, the human oxaliplatin-resistant gastric cancer cell line OCUM-2MD3/L-OHP was established, and the relationships between cell biological characteristics, the drug-resistant protein P-gp, and relationship between the apoptosis-suppressor gene livin and MDR in gastric cancer were investigated. Moreover, in vivo and in vitro anti-tumor effects and mechanisms of CIK combined with L-OHP on OCUM-2MD3/L-OHP cells were explored to provide experimental evidence for clinical application of CIK cells combined with chemotherapy in the treatment of drug-resistant gastric cancer.

## Materials

### Main instruments

The following instruments were used in this study: a -80°C ultra-low temperature refrigerator (SANYO, Japan), a -152°C Ultra-low temperature freezer (SANYO, Japan), an HT2 enzyme-linked immunosorbent assay (ELISA) reader (Anthos, Austria), an Epics-XL-II flow cytometer (Becoman Coulter, USA), a Diaphot 300 inverted phase contrast microscope (Nikon, Japan) and an H-7500 transmission electron microscope (Hitachi, Japan).

### Main reagents

The following reagents were used in this study: mouse-anti-human P-gp monoclonal antibody (ZSchem, Peking), rabbit-anti-human Livin monoclonal antibody (IMGENEX, USA), goat-anti-mouse fluorescent-labeled antibody and goat-anti-rabbit fluorescent-labeled antibody (Sino-American Biotech.).

### Cell culture

The human gastric cancer high invasion and metastasis cell line OCUM-2MD3 (parent cell line) was a gift from a professor in Surgical Department I of Osaka Medical University in Japan.

The human oxaliplatin-resistant gastric cancer high invasion and metastasis cell line OCUM-2MD3/L-OHP (resistant cell line) was constructed and cultured in our lab. The large dose (1.83 μg/ml) of L-OHP 24 h-repeated intermittent exposure method was applied as follows: DMEM medium containing L-OHP (1.83 μg/ml) was added to cells in logarithmic phase, fresh culture medium was replaced 24 h later, and this procedure was repeated until cells recovered growth. Death of the sensitive cells gradually appeared during induction, and the drug-resistant cells were grown continuously for six months. Cells were then cultured for two weeks with no drugs, IC_50 _values were gradually stabilized by detection of MTT (methyl thiazolyl tetrazolium) rapid colorimetry and cells were maintained in culture medium with no drugs. After cryopreservation and recovery of 10% DMSO culture medium, IC_50 _values were unchanged, indicating stabilization of drug resistance. All drug-resistance experiments were performed two weeks later in drug-free cultures. The two cell types were cultured in DMEM medium containing 10% fetal bovine serum, 100 μ/mL penicillin and 100 μ/mL streptomycin at 37°C in a humidified incubator containing 5% CO_2_. Cells in logarithmic phase were collected to prepare single-cell suspensions.

### Experimental drugs

The following experimental drugs were used in this study: L-OHP (Jiangsu Hengrui Medicine Co., Ltd.), 0.9% physiological saline diluted at concentrations of 1200 μg/mL, 600 μg/mL, 300 μg/mL, 150 μg/mL and 75 μg/mL, Irinotecan (IH), Gemcitabine (GEM) (IH and GEM obtained from Jiangsu Hengrui Medicine Co., Ltd.), cis-Diaminedichloroplatinum (CDDP), Carboplatin (CBDAC) (QILU Pharmaceutical Co., Ltd.), Mitomycin (MMC), Adriamycin (ADR) (MMC and ADR obtained from Zhejiang Hisun Pharmaceutical Co., Ltd.), Vincristine (VCR), Paclitaxel (PTX) (VCR and PTX obtained from Shanghai Hualian Pharmaceutical Factory) and 5-flurouracil (5-FU) (Shanghai Xudong Pharmaceutical Co., Ltd.).

### Effector cells

Preparation and in vitro amplification of CIK cells: The periphery heparin from healthy adults was obtained for anticoagulation, and prepared according to a previous report by Schmidt-Wolf IG et al. [17], cells were harvested in the 14^th ^day, and the ratio of potency and target was adjusted to 40:1, 20:1 or 10:1 before use.

### Construction and grouping of the human gastric cancer OCUM-2MD3/L-OHP cell peritoneal transplantation model

Preliminary experiments using our assay confirmed that the incidence of peritoneal tumors was 100% when each Balb/c nude mouse (female, 4~6 week, 15~18 g, animal licenses lot: SCXK 11-00-0005) was inoculated intraperitoneally with 5 × 10^6 ^drug-resistant cells. In our experiment, 35 nude mice were selected and inoculated intraperitoneally with drug-resistant cells at a dose of 5 × 10^6 ^cells per 0.2 ml each, and the human gastric cancer drug resistant cell peritoneal transplantation model was established. All mice were randomly divided into seven groups, including the normal control, NS control, L-OHP (1.125 mg/kg, 2.25 mg/kg), CIK (2 × 10^7^/0.2 mL, 4 × 10^7^/0.2 mL) and L-OHP+CIK groups. Intraperitoneal injection of drug-resistant cells was performed in the first six groups after 15 days of inoculation, once every other day for a total of three injection days. L-OHP (1.125 mg/kg) was administered to the L-OHP+CIK group after inoculation for 15 days, then CIK cells (2 × 10^7^/0.2 mL/number) were injected intraperitoneally twice every other day for a total of three injection days.

## Methods

### Observation of cell biological characteristics of OCUM-2MD3/L-OHP (Parental cells were used as control)

#### Cell morphology observation of drug-resistant cells

Both cell types were cultured on culture plates and observed under an inverted phase contrast microscope until the cells covered 80% of the bottom wall. Cells were collected (1 × 10^7 ^), fixed with 2.5% glutaraldehyde followed by 2% osmium tetroxide, dehydrated, embedded, sectioned, stained and observed and photographed with a transmission electron microscope.

#### Growth curve of OCUM-2MD3/L-OHP cells by cell count method

The two cell types were inoculated into 24-well plates at a density of 1.5 × 10^4 ^cells/well and cultured at 37°C in a humidified incubator containing 5% CO_2_. Three wells were used for live-cell counts each day, and a cell-growth curve was plotted after counting cells continuously for six days. Doubling time was calculated with the following equation: Td(h) = T × [log2/(logN-logN_0_)] (Td represents doubling time, T represents time in logarithmic proliferative phase (h), N represents cell numbers at the end of logarithmic proliferative phase, N_0 _represents cell numbers at the beginning of logarithmic proliferative phase).

#### Cell cycle distribution and apoptosis of drug-resistant cells analyzed by FCM (flow cytometry)

The two cell types (1 × 10^6^/ml) were collected, washed twice in PBS, fixed overnight with 70% cold ethanol and washed twice in PBS. Next, 10% chicken red blood cells were added as an internal control standard, 1 mL of propidium iodide (PI) (50 mg/L) was added, cells were kept at 4°C for 30 min, and FCM detection was performed after filtration by 500-mesh copper grid.

#### Detection of drug sensitivity in drug-resistant cells by MTT assay

##### Determination of sensitivity and resistance index (RI) of drug-resistant cells to L-OHP

A single-cell suspension of 5 × 10^4 ^cells/ml (200 μl/well) was added to a 96-well culture plate, and the culture medium containing L-OHP was added at final concentrations of 0.3, 0.6, 1.25, 2.5, 5, 10 and 20 μg/ml. Each concentration was tested in triplicate wells, and cells were cultured at 37°C in a humidified incubator containing 5% CO_2 _for 24 h. The supernatants were then discarded and 200 μl of serum-free medium and 20 μl of MTT (5 mg/L) were added in each well. Cells were cultured for 4 h, then supernatants were discarded, and 150 μl of DMSO was added to each well. The absorbance value of each well was measured by an automatic ELISA reader at a wavelength of 570 nm, and the inhibition rate and IC_50 _value of L-OHP at different concentrations were calculated according to the following equation: RI = IC50 (drug-resistant cell)/IC_50 _(parental cell).

##### Detection of MDR and cross resistance in drug-resistant cells

A single-cell suspension of 5 × 10^4 ^cells/ml (200 μl/well) was added to a 96-well culture plate, and the culture medium containing the chemotherapeutics L-OHP, CDDP, CBDCA, 5-Fu, ADM, MMC, GEM, VCR, IH and PTX were added at final concentrations of 5.4, 12.6, 695.0, 40.0, 6.2, 1.0, 66.0, 0.08, 72.9 and 11.6 μg/mL, respectively. Each drug was tested in triplicate. Cells were cultured at 37°C for 24 h in a humidified incubator containing 5% CO_2_, Supernatants were then discarded and 200 μl of serum-free medium and 20 μl of MTT (5 mg/L) were added to each well. Cells were cultured for 4 h, the supernatants were discarded, and 150 μl of DMSO was added in each well. The absorbance value of each well was measured by an automatic ELISA reader at a wavelength of 570 nm, the inhibition rate of each drug was calculated, and an inhibition rate less than 50% was set as the criteria for drug resistance.

#### Expression of P-gp and Livin in drug-resistant cells detected by FCM

The two cell types (each at a density of 1 × 10^6^/ml) were collected, washed in PBS twice, fixed overnight with 70% cold ethanol, and again washed in PBS twice. Cells were then incubated in 0.1 ml of mouse-anti-human P-gp and rabbit-anti-human Livin monoclonal antibodies at room temperature for 30 min and washed in PBS. The supernatants were discarded, 100 μl of goat-anti-mouse or goat-anti-rabbit fluorescent antibody was added according to the different detection index, incubated away from light at room temperature for 30 min, washed in PBS, and FCM detection was performed after filtration with a 500-mesh copper grid.

### Detection of in vitro killing activity by CIK combined with L-OHP on OCUM-2MD3/L-OHP cells

#### Groups (parent cells were set as controls for each group)

##### L-OHP intervention group

The in vitro killing activities of L-OHP applied alone at different concentrations against drug-resistant cells at 24 h, 48 h and 72 h were calculated.

##### CIK cell intervention group

The in vitro killing activities of CIK cells alone with different ratios of potency to target on drug-resistant cells were measured at 12 h, 24 h and 48 h.

##### CIK cell plus L-OHP intervention group

CIK cells with a ratio of potency to target of 40:1 were added for 12 h, and L-OHP at different concentrations was then added. The in vitro killing activities of combination of CIK and L-OHP applied in drug-resistant cells were measured 24 h later.

#### Detection of in vitro killing activity of L-OHP on drug-resistant cells

The two cell types (each at a density of 1 × 10^6^/ml) were collected and inoculated on 96-well plates (100 μl/well, 1 × 10^5 ^counts), and the drugs were added 24 h after cell adhesion.

L-OHP solutions were added (100 μl/well at final concentrations of 600, 300, 150, 75, and 37.5 μl/ml). The same volume of culture medium was added in the control group, and all treatments were tested in triplicate. Cells were cultured at 37°C in a humidified incubator containing 5% CO_2 _for 24 h, 48 h or 72 h, and 20 μl of MTT (5 mg/L) was then added to cultures.

Cells were cultured for 4 h then supernatants were discarded, and 150 μl of DMSO was added to each well. The absorbance value of each well was measured by an ELISA reader at a wavelength of 570 nm, and killing activity was calculated by the following equation from which IC_50 _values were calculated:

Killing activity (%) = (mean OD value in control group - mean OD value in experiment group) / (mean OD value in control group - mean OD value in blank control group) × 100%

#### Detection of in vitro killing activity of CIK on drug-resistant cells

The two cell types (each at a density of 1 × 10^6 ^cells/ml) were collected, inoculated in 96-well plates (100 μl/well, 1 × 10^5 ^cells), and CIK cells were added 24 h after cell adhesion.

CIK cells at different ratios of mixture Effector to Target (40:1, 20:1, 10:1) were added to a 96-well plate (100 μl/well). The same volume of culture medium was added in the control group, and blank control wells were also used. All treatments were tested in triplicate, and cells were cultured at 37°C in a humidified incubator containing 5% CO_2 _for 24 h, 48 h and 72 h. OD values were obtained by MTT assay with an automatic ELISA reader at a wavelength of 570 nm.

#### Detection of in vitro killing activity of CIK cells plus L-OHP on drug-resistant cells

CIK cells were added at an E to T ratio of 40:1 for 12 h. Various concentrations of L-OHP were then added, cells were cultured for 24 h continuously, and the killing activity of CIK and L-OHP was calculated.

### Anti-tumor effect of CIK plus L-OHP in the human drug-resistant gastric cancer cellular peritoneal transplantation model

Tumor weight and abdominal circumference were measured 21 days postinoculation (i.e., 7 days after intraperitoneal administration). The mice were sacrificed, and the number of ascites was calculated. The criterion for being cured was 60-day survival after inoculation with tumor cells.

#### Pathomorphological observations in the human drug-resistant gastric cancer cellular peritoneal transplantation model after the treatment of L-OHP and CIK cells

Tissue sections were acquired 24 h after final injection in each group, and macroscopic observation was used to detect changes of peritoneal transplantation nodules. The transplantation nodules in the omentum majus of each mouse were selected and divided into two sections, which were then used for routine pathological sectioning and transmission electron microscope examination.

### Statistical analyses

All data are expressed as mean ± SD, and analyses were carried out using SPSS 12.0 software (SPSS Inc, Chicago, IL). One-way analyses of variance (ANOVA), homogeneity tests for variance and Student's t-tests were used for comparisons of means. A p-value less than 0.05 was considered statistically significant.

## Results

### Cell biological characteristics of OCUM-2MD3/L-OHP cells

#### Morphological observations of drug-resistant cells

**Figure 1:**
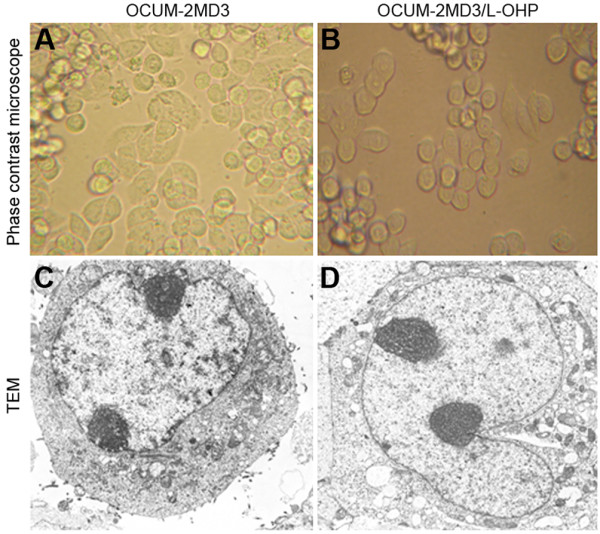
**A. OCUM-2MD3 cell (Phase contrast microscope × 400); B. OCUM-2MD3/L-OHP cell (Phase contrast microscope × 400); C. OCUM-2MD3 cell (TEM × 5000); D. OCUM-2MD3/L-OHP cell (TEM × 5000)**.

As is shown in Fig.1A and 1B, the two cell types in suspension appeared round under an inverted phase contrast microscope. Following cell adhesion, cells appeared spindle-shaped, were arranged in a single layer of different sizes, and showed no significant difference in cell morphology. The microvilli on the surface of parental cells were quite abundant under a transmission electron microscope, and the morphology of organelles in the cytoplasm was normal. The nuclei of the cells appeared abnormally large and were irregularly shaped. Moreover, euchromatin was abundant, heterochromatin was limited, and the nucleolus was large and clearly visible (Fig. 1C). There was no significant difference in morphology of drug-resistant cells compared with OCUM-2MD3 cells. (Fig. 1D).

#### Growth curve and population doubling time of drug-resistant cells

**Figure 2:**
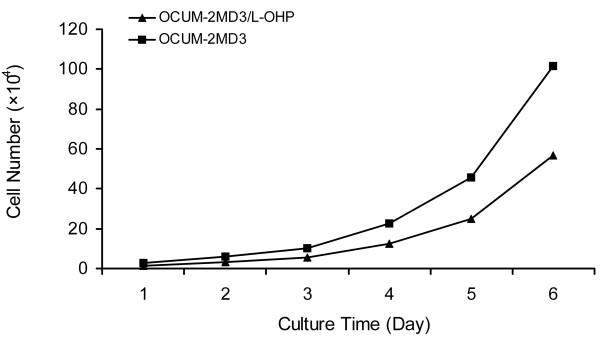
**Cell-growth curve of OCUM-2MD3/L-OHP**.

As shown in Fig. 2, proliferation speed of drug-resistant cells was slower than that of parental cells. The population doubling time of drug-resistant cells was 27.0 ± 2.04 h by cell counts, which extended for approximately 3 h (P < 0.05).

#### Cell cycle distribution and apoptosis of drug-resistant cells

**Figure 3:**
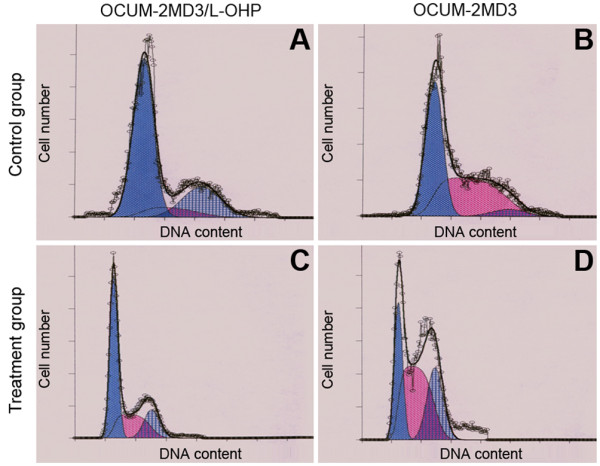
**Cell cycle**. (A). OCUM-2MD3/L-OHP (Control group); (B). OCUM-2MD3 (Control group); (C). OCUM-2MD3/L-OHP (Treatment group); (D). OCUM-2MD3 (Treatment group).

**Figure 4:**
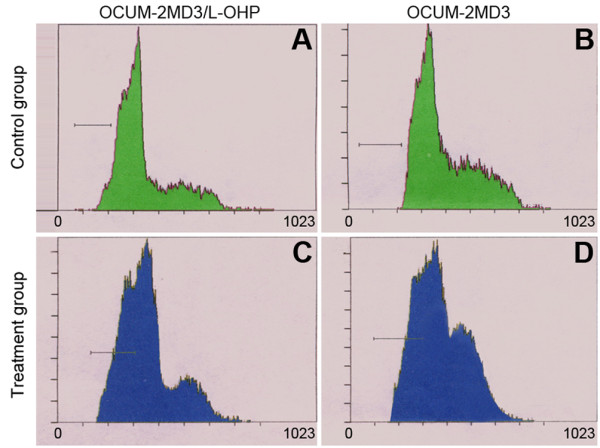
**Cell apoptosis**. (A). OCUM-2MD3/L-OHP (Control group); (B). OCUM-2MD3 (Control group); (C). OCUM-2MD3/L-OHP (Treatment group); (D). OCUM-2MD3 (Treatment group).

As shown in Table 1, Fig. 3 and Fig. 4, drug-resistant cell numbers in G_0_/G_1 _and G_2_/M phase were increased, whereas cell numbers in S phase were decreased, and the apoptosis rate was increased (P < 0.05). When cells were treated with L-OHP for 24 h, the drug-resistant cells in S phase increased in numbers, and parental cells in G_2_/M phase increased. That is, drug-resistant cells were arrested in G_2_/M phase by L-OHP, and parental cells were arrested in S phase. Meanwhile, apoptosis rates of both cell types were significantly enhanced, although the apoptosis rate in drug-resistant cells was less than the rate in parental cells (P < 0.05).

**Table 1:** Cell cycle distribution of OCUM-2MD3/L-OHP cells.

Cell	Cell cycle			Apoptosis rate (%)
		
	G_0_/G_1_	S	G_2_/M	
Control group				
OCUM-2MD3	47.93 ± 0.35	46.83 ± 2.31	5.22 ± 2.50	1.00 ± 0.11
OCUM-2MD3/L-OHP	66.03 ± 0.28*	10.4 ± 1.06*	23.25 ± 0.78*	5.21 ± 0.55*
Treatment group				
OCUM-2MD3	24.80 ± 0.52	49.37 ± 1.59	25.77 ± 1.30^Δ^	35.53 ± 0.73
OCUM-2MD3/L-OHP	50.80 ± 2.00	27.80 ± 0.86^Δ^	21.40 ± 2.79	29.43 ± 0.91*

* Comparisons of different cells in the same group P < 0.05

^Δ ^Comparisons of different cells in different groups P < 0.05

#### Sensitivity and RI of drug-resistant cells to L-OHP

**Figure 5:**
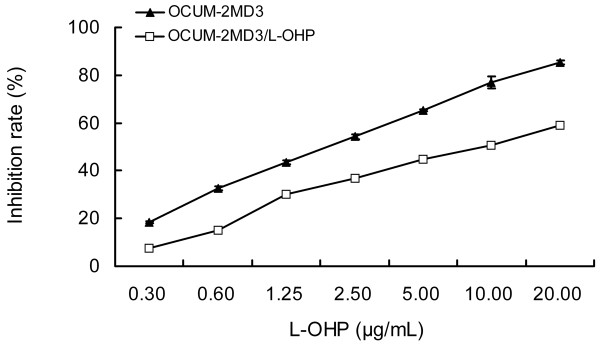
**Inhibition rate of various concentrations of L-OHP on drug-resistant cells**.

As shown in Fig. 5, with the rise of L-OHP concentration, inhibition rates of L-OHP on the two cell types gradually increased, and the inhibition rate of L-OHP on drug-resistant cells was significantly less than the inhibition rate of parental cells (P < 0.05). IC_50 _values of L-OHP on drug-resistant cells and parental cells at 24 h were 8.32 μg/mL and 1.92 μg/mL, respectively. In addition, the RI value of drug-resistant cells in response to L-OHP was 4.3. Following repeated passages, cryopreservation and recovery, the RI value remained stable.

#### Detection of MDR in drug-resistant cells

**Figure 6:**
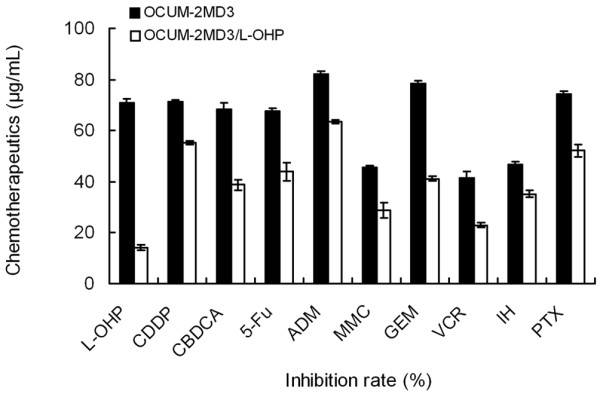
**Inhibition rates of different chemotherapeutics in drug-resistant cells**.

As is shown in Fig. 6, the inhibition rates of 10 chemotherapeutics, including L-OHP, CDDP, CBDCA, 5-Fu, ADM, MMC, GEM, VCR, IH and PTH, on drug-resistant cells were significantly less than inhibition rates in parental cells (P < 0.01). An inhibition rate less than 50% was set as the criterion for drug resistance, and parental cells showed drug resistance to MMC, VCR and IH. The drug-resistant cells were not only resistant to L-OHP, but their sensitivity to CDDP, ADM and PTX was also degraded and showed cross-resistance to CBDCA, 5-Fu, MMC, GEM, VCR and IH.

#### Expression of P-gp and Livin in drug-resistant cells

**Figure 7:**
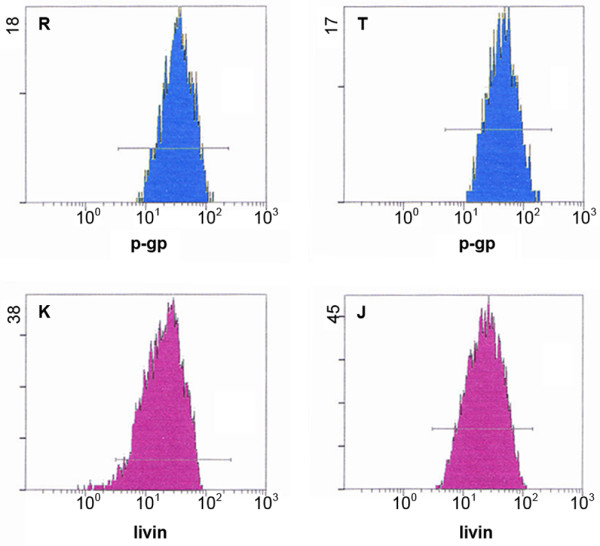
**Expression of P-gp and Livin in drug-resistant cells**. R: OCUM-2MD3 group; T: OCUM-2MD3/L-OHP group; K: OCUM-2MD3 group; J: OCUM-2MD3/L-OHP group.

As shown in Table 2 and Fig. 7, expression of P-gp and Livin was seen in both cell types. However, although P-gp expression in the drug-resistant cell line was higher than expression in the parental cells (P < 0.05), Livin expression showed no significant difference in two cell types (P > 0.05).

**Table 2:** Expression of P-gp and Livin in drug-resistant cells.

Cell line	Relative protein expression	
	
	P-gp	Livin
OCUM-2MD3	466.46 ± 12.04	467.82 ± 2.20
OCUM-2MD3/L-OHP	547.97 ± 7.76*	454.91 ± 8.56

* Compared with parental cell line, P < 0.05

### Detection of in vitro killing activity of CIK cells plus L-OHP on drug-resistant cells

#### In vitro killing activity of L-OHP on drug-resistant cells

As shown in Tables 3, 4, 5, resistances of drug-resistant cells to L-OHP increased 3.2-, 3.3- and 2.0-fold at the 24 h, 48 h and 72 h time points, respectively, when compared with the parental cells. The killing activity of L-OHP on drug-resistant cells and parental cells at 48 h was the most powerful, and killing activity increased with rising L-OHP concentrations.

**Table 3:** Cytotoxicity of L-OHP on OCUM-2MD3/L-OHP (μg/mL, %, $\bar{x}$ ± S, 24 h).

Group	600	300	150	75	37.5	IC50
OCUM-2MD3	76.2 ± 1.1	69.3 ± 2.3	57.7 ± 1.3	44.2 ± 0.9	28.3 ± 2.6	111.3
OCUM-2MD3/L-OHP	60.6 ± 0.5*	42.6 ± 1.3*	35.5 ± 4.2*	19.9 ± 1.7*	6.4 ± 2.1*	354.4

*Compared with OCUM-2MD3 Group P < 0.05

**Table 4:** Cytotoxicity of L-OHP on OCUM-2MD3/L-OHP (μg/mL, %, $\bar{x}$ ± *s*, 48 h).

Group	600	300	150	75	37.5	IC50
OCUM-2MD3	85.2 ± 0.9	74.6 ± 1.7	65.4 ± 2.1	51.2 ± 1.4	37.3 ± 2.2	71.2
OCUM-2MD3/L-OHP	72.4 ± 1.5*	52.7 ± 2.6*	43.5 ± 0.8*	26.4 ± 1.5*	9.8 ± 3.2*	235.2

*Compared with OCUM-2MD3 Group P < 0.05

**Table 5:** Cytotoxicity of L-OHP on OCUM-2MD3/L-OHP (μg/mL%, $\bar{x}$ ± S, 72 h).

Group	600	300	150	75	37.5	IC50
OCUM-2MD3	50.2 ± 1.8	40.6 ± 1.5	25.4 ± 2.7	19.2 ± 1.4	8.3 ± 1.7	522.3
OCUM-2MD3/L-OHP	38.4 ± 1.1*	24.7 ± 2.3*	17.5 ± 2.5*	9.8 ± 1.5*	5.6 ± 3.2*	1057.0

*Compared with OCUM-2MD3 Group P < 0.05

#### In vitro killing activity of CIK cells in drug-resistant cells

**Figure 8:**
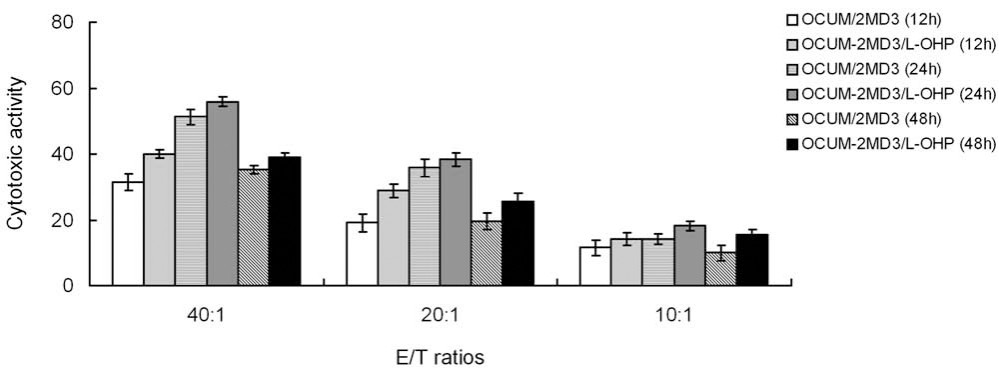
**Cytotoxic activity of CIK cells against tumor cells**.

As shown in Fig. 8, the killing activity of CIK cells on the two cell types peaked at 24 h and increased with the enhanced ratio of potency and target. Furthermore, the killing activity of CIK cells at each time point on drug-resistant cells were significantly higher than the killing activity of CIK cells on parental cells (P < 0.05). These findings suggest that CIK cells show more powerful in vitro killing activity on drug-resistant cells compared with the parental cells.

#### In vitro killing activity of CIK cells plus L-OHP in drug-resistant cells

As shown in Table 6, the in vitro killing activities of CIK cells combined with L-OHP in drug-resistant cells and parental cells were significantly enhanced when compared with L-OHP or CIK cells alone (P < 0.05), and killing activity was enhanced with the rise of L-OHP concentration. Compared with the parental cells, the in vitro killing activity of CIK cells plus L-OHP in drug-resistant cells showed greater synergetic lethal effects (P < 0.05).

**Table 6:** Cytotoxic activity of CIK cells plus L-OHP in OCUM-2MD3/L-OHP cells (μg/mL, %, $\bar{x}$ ± S).

Group	600	300	150	75	37.5	IC50
OCUM-2MD3	90.2 ± 1.7	81.1 ± 1.5	75.5 ± 2.9	65.3 ± 3.3	42.6 ± 1.6	44.5
OCUM-2MD3/L-OHP	94.5 ± 0.7*	85.0 ± 2.4*	79.4 ± 2.1*	67.7 ± 1.2*	50.9 ± 3.4*	36.8

*Compared with the OCUM-2MD3 group, P < 0.05

### Detection of in vivo activity of CIK cells plus L-OHP on drug-resistant cells

#### Effect of ascites and survival rate of L-OHP and CIK cells in the human gastric cancer resistant cellular peritoneal transplantation model

As shown in Table 7, survival rate for both the L-OHP group (1.125 mg/kg, 2.25 mg/kg) and the CIK group (2 × 10^7^/0.2 mL, 4 × 10^7^/0.2 mL) was significantly extended, and abdominal circumference was significantly reduced after treatment when compared with the NS control group (P < 0.01). Likewise, survival rate in the L-OHP plus CIK group was significantly further extended following treatment, and abdominal circumference was significantly further reduced compared with the NS control group (P < 0.01). Finally, there were no significant differences in either survival rate or abdominal circumference between the dual-treated group and the normal control group (P > 0.01).

**Table 7:** Effect on the model of gastric cancer by L-OHP, CIK, L-OHP+CIK ($\bar{x}$ ± S).

Group	n	Abdominal perimeter (cm)	Existed time (d)	Survival rate (35d)
Normal control group	5	8.8 ± 0.4	60 ± 0	5/5
NS control group	5	15.61 ± 0.5	20 ± 3.5	0/5
L-OHP1.125 mg/kg	5	14.45 ± 0.3^a^	38 ± 4.2^a^	3/5^a^
L-OHP2.25 mg/kg	5	12.15 ± 0.2^a^	52 ± 3.8^a^	4/5^a^
CIK2 × 10^7^/0.2 mL	5	13.90 ± 0.2^a^	40 ± 4.6^a^	3/5^a^
CIK4 × 10^7^/0.2 mL	5	11.87 ± 0.2^a^	53 ± 4.3^a^	4/5^a^
L-OHP+CIK	5	8.46 ± 0.3^ab^	60 ± 0^ab^	5/5^ab^

a) P < 0.01 Compared with NS control group

b) P > 0.01 Compared with normal control group

#### Pathomorphological effects of L-OHP and CIK cells in the human gastric cancer resistant cellular peritoneal transplantation model

##### Light microscope observations

**Figure 9:**
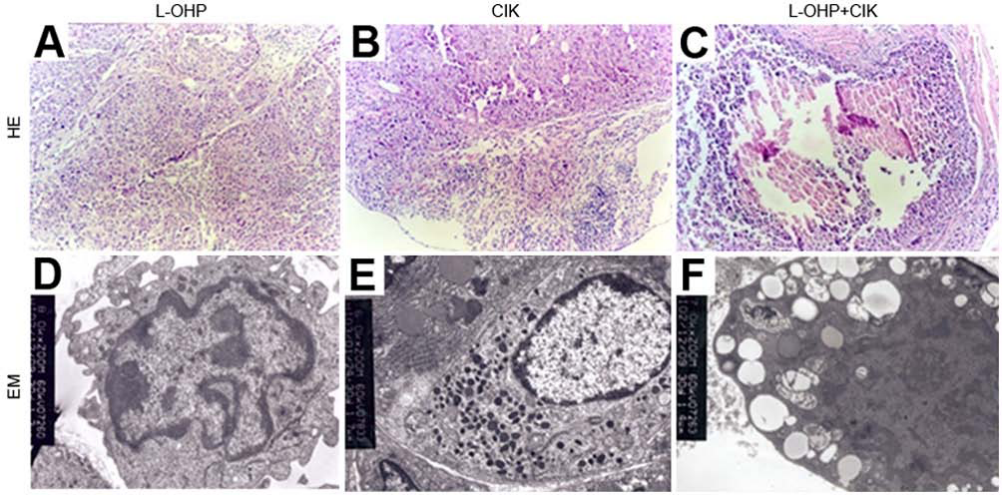
**Pathomorphological effects of L-OHP and CIK on the model of gastric cancer**. a) Effect of L-OHP (4.5 mg/kg, HE × 100) on the model of gastric cancer. b) Effect of CIK (4 × 10^7^/0.2 mL, HE × 100) on the model of gastric cancer. c) Effect of L-OHP+CIK (HE × 100) on the model of gastric cancer. d) Effect of L-OHP (2.25 mg/kg, EM, × 10.0 K) on the model of gastric cancer. e) Effect of CIK (2 × 10^7^/0.2 mL, EM, × 7.0 K) on the model of gastric cancer. f) Effect of L-OHP+CIK (EM, × 7.0 K) on the model of gastric cancer.

As shown in Fig. 9(a, b, c), the volume of cancer cells in the L-OHP group was reduced, and tumor hyperblastosis remained active. These data indicate that cell necrosis in the CIK cell group increased, and interstitial lymphocytes infiltrated. The cancer cell volume in the L-OHP+CIK group was significantly reduced, and a significant quantity of necrotic tissue and nested central necrosis were seen.

##### Transmission electron microscope observations

As shown in Fig. 9(d, e, f), the nuclear heterocytosome in the L-OHP group showed slight margination. In addition, after mixing cultures of CIK cells and tumor cells, pyknosis of tumor cell chromatin and nuclear margination appeared. Additionally, cytoplasmic swelling and severe vacuolar degeneration were seen in the L-OHP+CIK group. These findings suggest that cells in the L-OHP, CIK cell, and L-OHP+CIK group showed cancer cell necrosis, apoptotic changes and gradual aggravation.

## Discussion

Cell immunotherapy combined with chemotherapy for synergetic treatment of malignant tumors has been reported [18,19]. Although the 5-year survival rate for gastric cancer has improved, recurrence and metastasis remain the main factors affecting prognosis. Biochemical modulation is conducted as a novel therapeutic method applied in the clinic, whether this method increases the survival rate of gastric cancer patients is of most importance. Previous studies indicated that CIK combined with chemotherapy could provide a clinical benefit for gastric cancer patients by limiting progression [20,21], whereas studies on synergetic therapy for MDR tumors have reported quite limited outcome. The mechanism underlying the complementary killing effect of CIK cells combined with oxaliplatin in human gastric cancer resistant cells remains uncertain.

### Biological characteristic of OCUM-2MD3/L-OHP cells

The mechanism of drug resistance in tumor cells is quite complicated. Therefore, constructing an ideal drug-resistant cell line in vitro remains the premise and foundation for investigating drug-resistant mechanisms of tumor cells. Currently, there are only two methods for constructing drug-resistant tumor-cell lines available, including the drug concentration increment sustainable method and large dose medicine intermittent induction method. The method of gradually increasing drug-concentration in a culture medium is quite different from the repeated intermittent medication in clinical chemotherapy [22]. A recent study showed that when identical tumor cells were induced with the same drug at the same final concentration, but with different induction methods, drug-resistant cell lines with distinct drug-resistant mechanisms were produced [23]. The medication mode of large dose intermittent induction method mimics the processes seen in clinical chemotherapy. The drug-resistant cells induced with this method can maintain stable resistance and cell biological characteristics, even after being cultured for an extended duration in a drug-free culture medium. This feature is quite desirable for investigation of the drug-resistant cells.

In this study, we applied the IC_50 _concentration of L-OHP for 24 h (1.83 μg/ml) at the human gastric cancer cells according to the repeated intermittent exposure method and constructed oxaliplatin-resistant cell line OCUM-2MD3/L-OHP successfully. The RI of this cell line against L-OHP was 4.3, and after repeated passages, cryopreservation and recovery, the RI remained stable. According to Snow criteria [24], this cell line showed low drug resistance to L-OHP. The parental cells showed drug resistance to MMC, VCR and IH, showing characteristics of primary MDR. However, the induced drug-resistant cells are cross-resistant to CBDCA, 5-Fu, MMC, GEM, VCR and IH, but not L-OHP, showing features of secondary MDR. Additionally, there were no significant differences in morphology of the resistant cells compared with parental cells. In the resistant cells, the proliferation speed was slower, population doubling time was extended, and most cells were in G_0_/G_1 _phase. However, L-OHP only affects tumor cells from S phase to G_2_/M phase and may lead to attenuated chemotherapeutic sensitivities in resistant cells, which is possibly one of the mechanisms of secondary MDR.

The MDR gene MDR1 is located on 7q21.1 and encodes the P-gp protein as a transmembrane protein, which is composed of 1280 amino acid residues with a molecular weight of 170 kD. Twelve transmembrane domains and two ATP binding sites are located on the P-gp protein, which enable the molecule function as an energy-dependent drug-excretion pump, obstructing passive diffusion of drugs to the cytoplasm by activating an ATP pump. Additionally, P-gp can transport intracellular cytotoxic drugs outside of the membrane by active transport, leading to attenuation or deprivation of cytotoxic effects that generate the drug-resistance phenomenon and chemotherapeutic failure in the clinic [25]. The typical mechanism underlying MDR involves the MDR1 gene and overexpression of P-gp. P-gp overexpression was the most prominent drug-resistance mechanism generated in gastric cancer [26]. Our study indicates that P-gp is expressed both in drug-resistant cells and parental cells, and the expression of P-gp in drug-resistant cells was significantly higher than that in parental cells. Thus, we speculate that the secondary MDR was associated with upregulated P-gp expression, leading to drug resistance against L-OHP, CBDCA, 5-Fu, MMC, GEM, VCR and IH. The detection of P-gp expression levels in tumor tissues might help to choose optimized chemotherapeutic plan, reduce toxic side effects, and allow individualized chemotherapy.

Livin is a critical member of the apoptosis protein inhibitor family and binds caspases to inhibit their activity [27]. This effect causes cells to lose capability of programmed cell death, resulting in an imbalance of cell numbers in tissues and organs, and finally the formation of tumors. There is a critical correlation between the overexpression of livin and the impaired apoptosis mechanism in malignant tumor cells leading to apoptosis tolerance. In recent studies, Livin overexpression was found to be correlated with MDR mechanisms in multiple human tumors, such as leukemia, liver cancer and ovarian cancer [28-32]. In this study, we show that Livin expression was expressed in both drug-resistant cells and parental cells without significant difference, suggesting no direct correlation with mechanisms of secondary MDR.

### In vivo and in vitro killing activity of CIK cells plus L-OHP on OCUM-2MD3/L-OHP cells

Previous studies have shown that the overexpression of P-gp in MDR tumor cells enhances the immunogenicity of target cells, and makes the target cells more easily be recognized by immune effector cells. Therefore, the cytotoxic effect of immune effector cells against drug-resistant tumor cells was similar or even stronger than against parental cells. Moreover, maintenance of in vivo cytotoxicity against tumor cells was not necessarily dependent on the sustained administration of large doses of exogenous interleukin (IL)-2 [16,33-35]. Application of immunocytes, including CIK cells, may be a feasible treatment for drug-resistant tumors, although this treatment requires further investigation.

This study indicates that CIK cells manifeste stronger in vitro killing activity against drug-resistant cells than against parental cells. The possible mechanism underlying this phenomenon may be the CD3^+^CD56^+ ^double positive cells as cytoplasmic particles to kill tumor cells released when CIK cells are stimulated. Additionally, a large amount of inflammatory cytokines, such as TNF-α, IL-2 and GM-CSF, are released by the activated CIK cells, which can directly inhibit tumor cells, or indirectly kill tumor cells by modulating the immune system.

Previous studies suggested that CIK cells play a critical role in the accumulation of chemotherapeutic drugs in MDR tumor cells, and that the killing activity of CIK cells plus chemotherapeutic drugs against MDR tumor cells was significantly higher than with chemotherapeutic drugs along. Furthermore, the killing activity of CIK cells is proportional to the ratio of effector cells to target cells. However, the in vivo killing activity cannot be accurately measured [10]. Lack of this knowledge may result in unsatisfactory immune therapeutic effects in certain patients. The combination of immune effector cells and chemotherapeutic drugs against MDR target cells was able to improve the sensitivity of drug-resistant cells to chemotherapeutic drugs. This dual treatment showed excellent effects in scavenging remnant tumor cells expressing drug-resistant proteins in postoperative patients, even in drug-resistant tumors in middle and advanced stages irresponsible to radiotherapy and chemotherapy.

Our study revealed that the in vivo and in vitro killing activity of CIK cells combined with various concentrations of L-OHP against two types of tumor cells was significantly enhanced in comparison with the use of L-OHP or CIK cells alone. Moreover, the killing activity of CIK cells combined with L-OHP against drug-resistant cells showed stronger synergetic effects than the similar treatment of parental cells, providing evidence of improved anti-tumor effects for the clinical application of CIK cells combined with L-OHP.

## Competing interests

The authors declare that they have no competing interests.

## Authors' contributions

QZ conceived of the study, and participated in its design and coordination and draft the manuscript. HZ conceived of the study, and participated in producing CIK cells and helped to draft the manuscript. JL carried out the establishment of MDR cells, participated in the Observation of cell biological characteristics and helped to draft the manuscript. XH carried out the in vivo pharmacodynamics and pathomorphology experiments in vitro anti-tumor studies. YL and LF participated in the design of the study and performed the statistical analysis. All authors read and approved the final manuscript.

## References

[B1] Einhorn EH Testicular cancer: an oncological success story Clin Cancer Res 1997 3 2630 2632 10068265

[B2] Rixe O Ortuzar W Alvarez M Parker R Reed E Paull K Fojo T Oxaliplatin, tetraplatin, cisplatin, and carboplatin: spectrum of activity in drug-resistant cell lines and in the cell lines of the National Cancer Institute's Anticancer Drug Screen panel Biochem Pharmacol 1996 52 1855 1865 10.1016/S0006-2952(97)81490-6 8951344

[B3] Extra JM Espie M Calvo F Ferme C Mignot L Marty M Phase I study of oxaliplatin in patients with advanced cancer Cancer Chemother Pharmacol 1990 25 299 303 10.1007/BF00684890 2295116

[B4] Sanderson BJ Ferguson LR Denny WA Mutagenic and carcinogenic properties of platinum-based anticancer drugs Mutat Res 1996 355 59 70 8781577 10.1016/0027-5107(96)00022-x

[B5] Misset JL Bleiberg H Sutherland W Bekradda M Cvitkovic E Oxaliplatin clinical activity: a review Crit Rev Oncol Hematol 2000 35 75 93 10.1016/S1040-8428(00)00070-6 10936465

[B6] Cvitkovic E Ongoing and unsaid on oxaliplatin: the hope Br J Cancer 1998 77 Suppl 4 8 11 9647613 10.1038/bjc.1998.429 PMC2149881

[B7] Raymond E Faivre S Woynarowski JM Chaney SG Oxaliplatin: mechanism of action and antineoplastic activity Semin Oncol 1998 25 4 12 9609103

[B8] Chen CC Chen LT Tsou TC Pan WY Kuo CC Liu JF Yeh SC Tsai FY Hsieh HP Chang JY Combined modalities of resistance in an oxaliplatin-resistant human gastric cancer cell line with enhanced sensitivity to 5-fluorouracil Br J Cancer 2007 97 334 344 10.1038/sj.bjc.6603866 17609664 PMC2360324

[B9] Leemhuis T Wells S Scheffold C Edinger M Negrin RS A phase I trial of autologous cytokine-induced killer cells for the treatment of relapsed Hodgkin disease and non-Hodgkin lymphoma Biol Blood Marrow Transplant 2005 11 181 187 10.1016/j.bbmt.2004.11.019 15744236

[B10] Li HF Yang YH Shi YJ Wang YQ Zhu P Cytokine-induced killer cells showing multidrug resistance and remaining cytotoxic activity to tumor cells after transfected with mdr1 cDNA Chin Med J (Engl) 2004 117 1348 1352 15377427

[B11] Schmidt-Wolf IG Negrin RS Kiem HP Blume KG Weissman IL Use of a SCID mouse/human lymphoma model to evaluate cytokine-induced killer cells with potent antitumor cell activity J Exp Med 1991 174 139 149 10.1084/jem.174.1.139 1711560 PMC2118875

[B12] Lu PH Negrin RS A novel population of expanded human CD3+CD56+ cells derived from T cells with potent in vivo antitumor activity in mice with severe combined immunodeficiency J Immunol 1994 153 1687 1696 7519209

[B13] Scheffold C Brandt K Johnston V Lefterova P Degen B Schontube M Huhn D Neubauer A Schmidt-Wolf IG Potential of autologous immunologic effector cells for bone marrow purging in patients with chronic myeloid leukemia Bone Marrow Transplant 1995 15 33 39 7538001

[B14] Verneris MR Kornacker M Mailander V Negrin RS Resistance of ex vivo expanded CD3+CD56+ T cells to Fas-mediated apoptosis Cancer Immunol Immunother 2000 49 335 345 10.1007/s002620000111 10946816 PMC11037019

[B15] Kim HM Kang JS Lim J Park SK Lee K Yoon YD Lee CW Lee KH Han G Yang KH Kim YJ Kim Y Han SB Inhibition of human ovarian tumor growth by cytokine-induced killer cells Arch Pharm Res 2007 30 1464 1470 10.1007/BF02977372 18087816

[B16] Schmidt-Wolf IG Lefterova P Johnston V Scheffold C Csipai M Mehta BA Tsuruo T Huhn D Negrin RS Sensitivity of multidrug-resistant tumor cell lines to immunologic effector cells Cell Immunol 1996 169 85 90 10.1006/cimm.1996.0094 8612299

[B17] Schmidt-Wolf IG Lefterova P Mehta BA Fernandez LP Huhn D Blume KG Weissman IL Negrin RS Phenotypic characterization and identification of effector cells involved in tumor cell recognition of cytokine-induced killer cells Exp Hematol 1993 21 1673 1679 7694868

[B18] Wu C Jiang J Shi L Xu N Prospective study of chemotherapy in combination with cytokine-induced killer cells in patients suffering from advanced non-small cell lung cancer Anticancer Res 2008 28 3997 4002 19192663

[B19] Shi M Yao L Wang FS Lei ZY Zhang B Li WL Liu JC Tang ZR Zhou GD [Growth inhibition of human hepatocellular carcinoma xenograft in nude mice by combined treatment with human cytokine-induced killer cells and chemotherapy] Zhonghua Zhong Liu Za Zhi 2004 26 465 468 15555334

[B20] Toge T Effectiveness of immunochemotherapy for gastric cancer: a review of the current status Semin Surg Oncol 1999 17 139 143 10.1002/(SICI)1098-2388(199909)17:2<139::AID-SSU9>3.0.CO;2-R 10449686

[B21] Jiang J Xu N Wu C Deng H Lu M Li M Xu B Wu J Wang R Xu J Nilsson-Ehle P Treatment of advanced gastric cancer by chemotherapy combined with autologous cytokine-induced killer cells Anticancer Res 2006 26 2237 2242 16821594

[B22] Liang Z Bian D Experimental study on the mechanism of cisplatin resistance and its reversion in human ovarian cancer Chin Med J (Engl) 1996 109 353 355 9208490

[B23] Yang LY Trujillo JM Biological characterization of multidrug-resistant human colon carcinoma sublines induced/selected by two methods Cancer Res 1990 50 3218 3225 2334917

[B24] Snow K Judd W Characterisation of adriamycin- and amsacrine-resistant human leukaemic T cell lines Br J Cancer 1991 63 17 28 1989661 10.1038/bjc.1991.7 PMC1971666

[B25] Gottesman MM Pastan I Biochemistry of multidrug resistance mediated by the multidrug transporter Annu Rev Biochem 1993 62 385 427 10.1146/annurev.bi.62.070193.002125 8102521

[B26] Zheng G Han F Liu X [Drug resistance mechanism of doxorubicin-resistant human gastric cancer cells BGC-823/DOX] Zhonghua Wai Ke Za Zhi 1997 35 325 328 10374463

[B27] Scott FL Denault JB Riedl SJ Shin H Renatus M Salvesen GS XIAP inhibits caspase-3 and -7 using two binding sites: evolutionarily conserved mechanism of IAPs EMBO J 2005 24 645 655 10.1038/sj.emboj.7600544 15650747 PMC548652

[B28] Qiuping Z Jie X Youxin J Qun W Wei J Chun L Jin W Yan L Chunsong H Mingzhen Y Qingping G Qun L Kejian Z Zhimin S Junyan L Jinquan T Selectively frequent expression of CXCR5 enhances resistance to apoptosis in CD8(+)CD34(+) T cells from patients with T-cell-lineage acute lymphocytic leukemia Oncogene 2005 24 573 584 10.1038/sj.onc.1208184 15580304

[B29] Ganten TM Koschny R Haas TL Sykora J Li-Weber M Herzer K Walczak H Proteasome inhibition sensitizes hepatocellular carcinoma cells, but not human hepatocytes, to TRAIL Hepatology 2005 42 588 597 10.1002/hep.20807 16037944

[B30] Moriai R Asanuma K Kobayashi D Yajima T Yagihashi A Yamada M Watanabe N Quantitative analysis of the anti-apoptotic gene survivin expression in malignant haematopoietic cells Anticancer Res 2001 21 595 600 11299811

[B31] Yan XJ Liang LZ Zeng ZY Shi Z Fu LW [Effect of survivin shRNA on chemosensitivity of human ovarian cancer cell line OVCAR3 to paclitaxel] Ai Zheng 2006 25 398 403 16613669

[B32] Zaffaroni N Pennati M Colella G Perego P Supino R Gatti L Pilotti S Zunino F Daidone MG Expression of the anti-apoptotic gene survivin correlates with taxol resistance in human ovarian cancer Cell Mol Life Sci 2002 59 1406 1412 10.1007/s00018-002-8518-3 12363043 PMC11337548

[B33] Azuma E Masuda S Qi J Kumamoto T Hirayama M Nagai M Hiratake S Umemoto M Komada Y Sakurai M Cytotoxic T-lymphocytes recognizing P-glycoprotein in murine multidrug-resistant leukemias Eur J Haematol 1997 59 14 19 10.1111/j.1600-0609.1997.tb00954.x 9260576

[B34] Arienti F Gambacorti-Passerini C Borin L Rivoltini L Orazi A Pogliani EM Corneo G Parmiani G Increased susceptibility to lymphokine activated killer (LAK) lysis of relapsing vs. newly diagnosed acute leukemic cells without changes in drug resistance or in the expression of adhesion molecules Ann Oncol 1992 3 155 162 1606087 10.1093/oxfordjournals.annonc.a058133

[B35] Margolin KA Wright C Forman SJ Autologous bone marrow purging by in situ IL-2 activation of endogenous killer cells Leukemia 1997 11 723 728 10.1038/sj.leu.2400646 9180298

